# Factors Associated with Hospital Length of Stay and Interhospital Transfer in Patients with Rib Fractures: A Retrospective Study Using the Japan Trauma Data Bank

**DOI:** 10.3390/jcm15218728

**Published:** 2026-11-09

**Authors:** Keisuke Motohama, Wataru Ishii, Mami Nakamura, Kosai Cho, Masahito Hitosugi

**Affiliations:** 1 Department of Legal Medicine, Shiga University of Medical Science, Shiga 520-2192, Japan; mamin@belle.shiga-med.ac.jp (M.N.); hitosugi@belle.shiga-med.ac.jp (M.H.); 2 Critical Care Center, Emergency Medicine, Kyoto Medical Center, Kyoto 612-8555, Japan; kosai.cho@gmail.com; 3 Critical Care Center, Emergency Medicine, Kyoto Daini Red Cross Hospital, Kyoto 602-8026, Japan; wataruaug0804@lily.ocn.ne.jp

**Keywords:** rib fracture, hospital length of stay, interhospital transfer, Japan Trauma Data Bank, comorbidity

## Abstract

**Background/Objectives**: Patient characteristics and concomitant injuries may influence hospital length of stay (LOS) and discharge disposition after rib fractures. We examined these associations in a selected Japanese trauma-registry population. **Methods**: This retrospective study used Japan Trauma Data Bank records from 2019–2021. Adult survivors with target rib-fracture Abbreviated Injury Scale (AIS) codes, no additional recorded chest AIS codes, and maximum extra-chest AIS < 2 were included; recorded chest compressions were excluded. The one- or two-rib and three-or-more-rib groups were analyzed separately using negative binomial regression for recorded index-hospital LOS and Firth logistic regression for transfer versus home discharge. **Results**: Eligible samples comprised 149 and 787 patients; updated complete-case LOS samples were 126 and 694, and transfer samples were 142 and 702. In the linear-summary models, the adjusted LOS ratio for two versus one fractured rib was 1.936 (95% CI, 1.227–3.055). In the larger cohort, the ratios were 1.231 (1.156–1.310) per 10 years of age and 0.796 (0.722–0.879) per Glasgow Coma Scale (GCS) point. Adjusted transfer odds ratios per Charlson Comorbidity Index (CCI) point were 2.630 (1.531–4.960) and 1.278 (1.109–1.479). Flexible-age analyses indicated nonlinearity for LOS in the smaller cohort and transfer in the larger cohort. Some estimates changed with eligibility criteria and model specification. **Conclusions**: Associations with recorded LOS and interhospital transfer were observed among selected survivors, but their interpretation is limited by selection, incomplete data, unmeasured institutional and functional characteristics, and model sensitivity. These exploratory results do not establish causal effects or validated clinical decision rules.

## 1. Introduction

Rib fractures are common in trauma care and are associated with pulmonary complications, longer hospitalization, and mortality [1,2]. Older patients have higher risks of pneumonia and death [3,4,5], and associations between the number of fractured ribs and clinical outcomes have also been investigated [6,7]. A practice management guideline has evaluated nonsurgical management and analgesia in older adults with multiple rib fractures [8]. A systematic review of older patients also highlights the difficulty of interpreting treatment outcomes when injury severity and concomitant injuries differ between groups [9]. Patients with rib fractures are heterogeneous in terms of injury mechanism, concomitant injuries, and fracture pattern; assessment therefore requires consideration of associated thoracic injuries as well as fracture number [10,11].

Hospital length of stay (LOS) and discharge disposition are important outcomes alongside mortality and pulmonary complications. Halevi et al. reported associations of age, fracture number, and comorbidities with discharge to a destination other than home among older patients with rib fractures [12]. Comparisons of trauma registries in Japan and the United States have shown differences in LOS and discharge disposition, supporting the value of evaluating these outcomes in the Japanese healthcare setting [13]. Associations between injury severity, hospital stay, and resource use have also been examined using the JTDB [14].

Previous JTDB studies have addressed surgical rib fixation, isolated chest trauma, and composite adverse outcomes [15,16,17]. Examining recorded LOS and interhospital transfer separately within common, restrictive injury criteria may provide complementary evidence in the Japanese healthcare setting. This is an incremental contribution rather than identification of previously unknown risk factors.

We therefore examined factors associated with recorded index-hospital LOS and interhospital transfer among selected adult survivors with target AIS 1–3 rib-fracture codes, no additional recorded chest AIS codes, and no extra-chest AIS ≥2 injuries. We analyzed patients with one or two rib fractures separately from those with three or more.

## 2. Materials and Methods

### 2.1. Study Design and Data Source

This retrospective observational study used JTDB records from 2019–2021. The JTDB is a nationwide multicenter trauma registry in Japan, established through a joint initiative of the Japanese Association for Acute Medicine and the Japanese Association for the Surgery of Trauma to collect and analyze trauma data and improve the quality of trauma care. It records patient characteristics, injury mechanisms, physiological findings on arrival, anatomical and physiological injury severity, prehospital and in-hospital interventions, and clinical outcomes at participating institutions [18].

### 2.2. Study Population and Selection

We identified patients with AIS 2008 codes of 450201.1 (one rib fracture; AIS 1), 450202.2 (two rib fractures; AIS 2), or 450203.3 (three or more rib fractures; AIS 3). The same AIS 2008 codes were used across all study years. Within each fracture category, patients were excluded if they had any AIS code beginning with 4 other than the target rib fracture code.

We further excluded patients younger than 18 years or with missing age. Patients who underwent chest compressions during resuscitation were excluded because the procedure itself can cause rib fractures. Patients who died were excluded because the available JTDB data did not permit identification of the cause of death or differentiation between trauma-related and non-trauma-related deaths. Furthermore, patients with a maximum AIS score of 2 or higher in any extra-chest region were excluded. The eight extra-chest regions were the head, face, neck, abdomen, spine, upper extremities, lower extremities, and external region. Registry data were used to determine resuscitation procedures and mortality.

The same AIS 2008 code definitions were used throughout 2019–2021. No patient carried more than one distinct target rib-fracture code in the checked data; repeated entries of the same code did not create additional patients. The three-or-more-rib code did not provide an exact fracture count.

These criteria were used to minimize the influence of other injuries and identify patients whose principal injury was a rib fracture classified as AIS 1–3.

Considering the sample sizes in the individual categories, patients with one and two rib fractures were combined into a one- or two-rib group. This group and the three-or-more-rib group were analyzed separately.

### 2.3. Outcomes and Explanatory Variables

The outcomes were registry-recorded LOS in days and discharge disposition. LOS was analyzed without dichotomization; zero-day records were retained. Missing or negative LOS was excluded from LOS models. We used the recorded field rather than reconstructing elapsed time from timestamps. The exact registry convention for partial days was not independently verified; a zero-day record was not assumed to establish a stay shorter than 24 h. Hospitalization at referring and receiving institutions was not linked or added.

Discharge analyses included patients with confirmed discharge home or transfer to another medical institution. Home discharge was the reference outcome, and transfer was the event. Records classified as other, unknown, or missing were excluded from this comparison. Missing or invalid LOS alone was not a reason for exclusion from discharge analyses.

Patient characteristics included age, sex, injury mechanism, fracture number, and CCI. We calculated CCI from the original 19 component conditions and weights [19], without age points; age was entered separately. Blank or unknown individual conditions contributed no points, which does not confirm clinical absence. A missing total CCI was not replaced with zero.

Injury mechanism was classified as traffic injury, falls (including same-level falls and other recorded fall categories), or other/unclassifiable. The last category combined other recorded mechanisms and unavailable mechanism information; these components were counted separately in sensitivity assessments. Findings on arrival included blood pressure, heart rate, respiratory rate, SpO_2_, total GCS, and oxygen administration. Recorded unknown flags took precedence over numerical measurements. RR ≥60 breaths/min was treated as invalid for analysis, and total GCS outside 3–15 was unavailable; GCS was not reconstructed from its components.

### 2.4. Descriptive Statistics and Comparisons by Discharge Disposition

Continuous variables were summarized as means and standard deviations and categorical variables as counts and percentages. Percentages were calculated using the number of nonmissing observations for each variable as the denominator, and missing counts were reported.

Unadjusted comparisons between home discharge and transfer used Welch’s t-test for continuous variables and Fisher’s exact test for categorical variables. Missing or unknown values were excluded separately for each variable. Injury mechanism was tested as a three-category variable. Statistical significance in these comparisons was not used as the criterion for selecting explanatory variables for multivariable analyses.

### 2.5. Multivariable Analysis of LOS

Negative binomial regression with a log link was used for LOS. We used NB2 parameterization, Var(Y|X) = μ + αμ^2^. The one- or two-rib model included age, injury mechanism, fracture number, CCI, systolic blood pressure, respiratory rate, and total GCS. Fracture number was omitted in the three-or-more-rib model because its exact value was unavailable. SBP, RR, and GCS were selected with reference to the physiological components of the Revised Trauma Score; the RTS itself was neither calculated nor entered, and its coding and weights were not applied.

Combining one and two fractures was supported by their shared Grade I category for closed fractures involving fewer than three ribs in the AAST chest wall Organ Injury Scale [20], as well as the small individual samples. This does not establish equivalent outcomes; the combined LOS model explicitly adjusted for two versus one fractured rib.

Complete-case analysis was used without imputation. Results are adjusted mean LOS ratios with model-based Wald 95% CIs and two-sided Wald *p*-values, scaled per 10 years of age, 10 mmHg SBP, 5 breaths/min RR, and 1 CCI or GCS point. Mechanism contrasts are traffic injury versus falls and versus other/unclassifiable; fracture number is two versus one. These are mutually adjusted associations, not causal effects or ratios of median LOS.

We assessed Poisson overdispersion, NB dispersion, observed versus fitted zero counts, and sensitivity to long stays. Additional analyses relaxed maximum extra-chest AIS to ≤2, excluded zero-day LOS, excluded the longest stay or values above the within-group 99th percentile, and used a Poisson log-link mean model with HC1 sandwich covariance. These alternative standard errors address variance misspecification, not hospital clustering. GCS < 15 versus 15 and three-knot restricted cubic splines for age, SBP, and RR assessed functional form. Knots were at the 10th, 50th, and 90th percentiles of each analytical sample. Linear-age primary models were retained as reference analyses; spline results were reported separately rather than replacing models based only on a significance test.

### 2.6. Multivariable Analysis of Transfer

Firth penalized logistic regression included age, injury mechanism, and CCI. This limited complexity with only 23 transfer events in the smaller cohort but did not adjust for fracture number in that model. Coefficients were estimated by penalized maximum likelihood; 95% CIs used penalized profile likelihood and two-sided *p*-values used penalized likelihood-ratio tests. These specifications were developed during analysis, not prospectively prespecified.

LOS was not included as a covariate in the multivariable transfer models because LOS is an in-hospital course variable that is temporally and structurally related to discharge disposition.

Complete cases with confirmed home discharge or interhospital transfer were analyzed; the latter was the event. Adjusted odds ratios are not risk ratios or proportional changes in transfer probability. We assessed leave-one-out coefficient sensitivity in the smaller cohort and age nonlinearity in the larger cohort. Additional year-adjusted and mechanism-coding analyses used the same primary covariates; details are in the supplement.

All analyses were exploratory, with two-sided numerical *p*-values and no multiplicity adjustment. We emphasize effect sizes, CIs, and sensitivity rather than a 0.05 threshold. Separate group analyses did not test between-group interactions. Updated analyses used R 4.5.1, MASS 7.3–65, and logistf 1.26.1; their pre-cleaning estimates reproduced the prior JMP Pro 18 tables to three decimal places. Hospital identifiers were unavailable, precluding hospital-level adjustment.

### 2.7. Ethical Considerations

The JTDB data were anonymized. Ethical review and approval and individual informed consent were not required under the applicable institutional framework. This study was conducted in accordance with the Declaration of Helsinki.

### 2.8. Use of Generative Artificial Intelligence

During the preparation of this manuscript, the authors used ChatGPT (GPT-6.1 Sol, OpenAI) to assist with refinement of the English text. The authors reviewed and edited the output and take full responsibility for the content of this publication.

## 3. Results

### 3.1. Study Population

**Figure 1:**
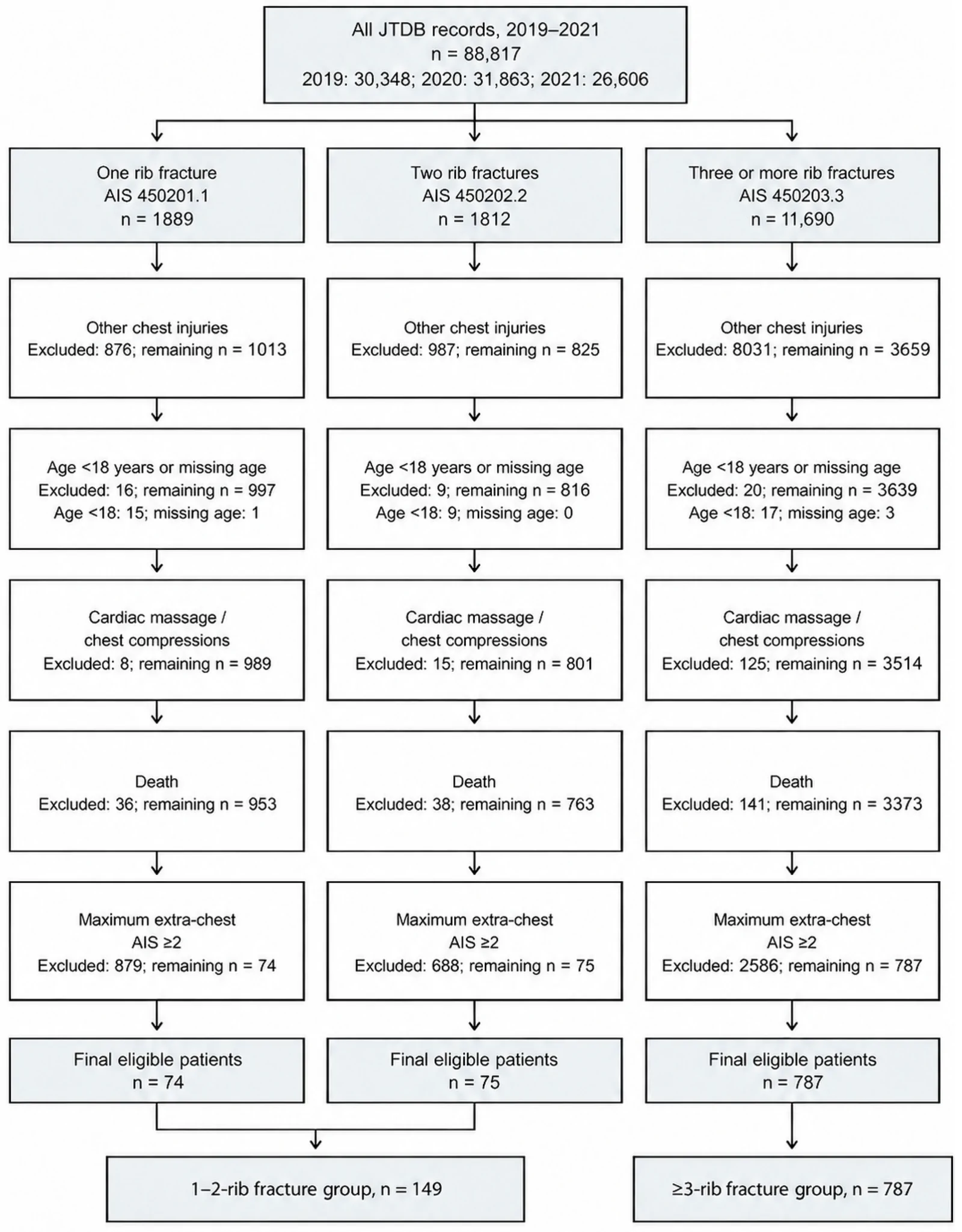
Patient selection flowchart. The final study population comprised 936 patients. Other chest injury exclusions mean any additional recorded AIS code beginning with 4, other than the category-specific target code. The diagram describes coded eligibility rather than independent clinical verification of every possible chest injury. Extra-chest maximum AIS was assessed across eight regions; missing age was excluded with age <18 years.

The final eligible population comprised 936 patients: 74 with one rib fracture, 75 with two, and 787 with three or more. The combined groups contained 149 and 787 patients. Figure 1 shows sequential selection from 88,817 registry records. At the mortality-exclusion step, before the extra-chest AIS restriction, 36, 38, and 141 deaths were excluded from the respective fracture categories.

LOS was unavailable for 9/149 and 43/787 patients (including one negative value in the larger group). Of the remaining 140 and 744, covariate incompleteness excluded 14 and 50, leaving 126 and 694. Within patients with valid LOS, missing SBP/RR/GCS counts were 1/9/8 and 7/32/20; reasons overlap. The larger sample decreased from 696 to 694 after RR cleaning. Zero-day LOS occurred in 20 and 114 eligible patients, including 19/126 (15.1%) and 95/694 (13.7%) complete cases.

Discharge disposition in the one- or two-rib group was home for 119 patients, another medical institution for 23, other for two, and missing for five. Corresponding counts in the three-or-more-rib group were 545, 158, 13, one unknown, and 70 missing. Patient characteristics and outcomes are presented in Table 1, and patient selection in Figure 1.

### 3.2. Factors Associated with LOS

The one- or two-rib LOS model included 126 complete cases (59 with one fracture and 67 with two). The updated three-or-more-rib model included 694. These linear-summary reference models are presented in Table 2.

In the smaller cohort, the adjusted mean LOS ratios were 1.166 (95% CI, 1.013–1.343) per 10 years of age, 1.936 (1.227–3.055) for two versus one fractured rib, and 1.107 (1.019–1.203) per 10 mmHg SBP. The age summary does not imply a uniform association across the age range.

Traffic-injury contrasts were 0.535 (0.313–0.912) versus falls and 0.425 (0.213–0.849) versus other/unclassifiable mechanisms. CCI, RR, and GCS estimates had CIs including 1.

In the larger cohort, ratios were 1.231 (1.156–1.310) per 10 years of age and 0.796 (0.722–0.879) per GCS point. CIs for injury mechanism, CCI, SBP, and RR included 1. Concentration of GCS near 15 limits a constant per-point interpretation.

Sensitivity analyses were not uniformly consistent. With expanded eligibility, the smaller-cohort fracture-number ratio was 0.967 (0.805–1.162), compared with 1.936 in the primary model. Excluding the longest LOS increased it to 2.476 (1.597–3.839), whereas the alternative Poisson-HC1 model gave 1.418 (0.662–3.037). In the larger cohort, age and GCS directions were retained across these assessments. Full results appear in Supplementary Tables S3–S5 and S9–S11.

### 3.3. Unadjusted Comparisons by Discharge Disposition

In the smaller cohort, 119 home discharges and 23 transfers were compared. Mean ages were 57.0 and 75.7 years; mean CCI values were 0.3 and 1.4. Tables S1 and S2 give variable-specific denominators and exact numerical *p*-values.

In the larger cohort, 545 home discharges and 158 transfers were compared. Mean ages were 66.2 and 75.1 years, and CCI values were 0.5 and 1.1. These unadjusted comparisons do not determine model inclusion.

The recorded mechanism distribution differed between disposition groups. Physiological summaries use their own available-case denominators; GCS and SpO_2_ were lower on average among transferred patients. These findings do not establish the reason for transfer or a causal role of physiological measurements.

### 3.4. Factors Associated with Transfer

The smaller transfer model included 119 home discharges and 23 transfers. Linear-summary adjusted odds ratios were 1.476 (1.061–2.139) per 10 years of age and 2.630 (1.531–4.960) per CCI point.

The larger model included 544 home discharges and 158 transfers (702 total); one home discharge with missing CCI was excluded from the 703 confirmed dispositions. Linear-summary odds ratios were 1.353 (1.185–1.554) per 10 years of age and 1.278 (1.109–1.479) per CCI point. However, flexible-age assessment indicated nonlinearity in this cohort, so the age ratio is not assumed constant across ages. Table 3 provides all estimates.

### 3.5. Additional Assessments

Poisson Pearson χ^2^/df values were 11.08 and 34.11, compared with 1.38 and 2.68 for NB models; estimated NB α values were 1.160 and 1.345. Predicted zero counts (30.5/125.9) exceeded observed counts (19/95), indicating imperfect fit (Table S8). After zero-day exclusion, the smaller-cohort age ratio was 1.120 (0.983–1.277); the larger-cohort age/GCS directions persisted (Table S9).

GCS was 15 in 101/126 and 547/694 cases. Replacing continuous GCS with <15 versus 15 gave ratios of 1.048 (0.617–1.778) and 1.924 (1.542–2.400), respectively (Table S12). Spline nonlinearity *p*-values were 0.0035 for smaller-cohort LOS, 0.809 for larger-cohort LOS, and 0.0036 for larger-cohort transfer. The flexible models yielded age-80-versus-60 ratios of 2.171 (1.439–3.277), 1.529 (1.323–1.768), and 2.061 (1.579–2.692), respectively, with pointwise Wald CIs (Tables S13 and S14; Figure S1). These are age-specific contrasts, not uniform 10-year effects.

Included/excluded comparisons and mechanism components are in Tables S6, S7 and S15. Splitting falls in the larger cohort, excluding unavailable mechanisms, and year adjustment produced broadly similar key estimates (Tables S16–S18), without eliminating residual confounding. Leave-one-out smaller-cohort transfer estimates ranged from 1.393–1.606 for age and 2.440–3.396 for CCI (Table S19); these ranges are not CIs. Sequential selection and coding definitions are in Table S20 and the reproducibility materials.

## 4. Discussion

This study examined recorded index-hospital LOS and transfer in selected adult survivors from a multicenter Japanese registry. Some estimates depended on eligibility, observations, and model specification. Flexible age results preclude uniform per-10-year interpretations in two models. Findings are exploratory associations, not causal effects or validated predictions.

### 4.1. Comparison with Previous Studies and Contribution of This Study

Prior studies describe age, fracture burden, and comorbidity associations with pneumonia, mortality, and hospitalization [3,4,7,21]. Our study adds incremental evidence on two separately defined hospital outcomes in a restrictive registry population; differing populations and outcomes limit direct comparisons.

Kishawi et al. assessed a composite including death, pneumonia, tracheostomy, and prolonged hospitalization [17]. Our separate outcomes and survivor restriction address a narrower question. This is not identification of previously unknown risk factors.

Halevi et al. examined discharge to a facility [12], whereas our outcome is transfer to another medical institution. Differences in destination definitions and care organization limit direct comparisons. Neither outcome is necessarily a direct measure of functional dependence.

Japan–US registry comparisons provide healthcare-system context [13,22], not evidence that our selected cohort represents all Japanese rib-fracture patients. Registry participation and local pathways can shape recorded LOS and disposition.

### 4.2. Factors Associated with LOS

Physical reserve, frailty, function, and social circumstances may influence hospital course. Frailty studies [23,24] reinforce that age, comorbidity, and frailty are distinct. Our data did not adequately capture these differences.

The smaller-cohort fracture-number association persisted after long-stay exclusions but attenuated with expanded injury eligibility and alternative estimation. This demonstrates population and model dependence, not equivalence of one and two fractures. The three-or-more-rib category also remains heterogeneous because exact counts were unavailable.

GCS was concentrated near 15; categorical sensitivity supports a lower-GCS association in the larger cohort but not a constant per-point effect across its full range. Admission measurements may reflect prior treatment or sedation. Recorded LOS is not total hospitalization: transfer arrangements may lengthen the index stay, while early transfer may shorten it despite continued inpatient care. LOS and transfer therefore reflect overlapping processes.

### 4.3. Patient Characteristics Associated with Transfer and Clinical Implications

CCI summarizes recorded conditions but does not measure frailty, physical function, or all baseline health differences. Unavailable component conditions contributed no points and may underestimate burden. Equal CCI can represent different disease combinations. Research on complications after chest trauma [25] does not directly explain transfer.

Age and CCI associations may partly reflect unmeasured functional, cognitive, and social characteristics. Individual disease effects cannot be inferred from the aggregate CCI. Transfer denotes a recorded destination only; the receiving institution’s function and clinical reason were unavailable.

Transfer cannot be equated with newly developed disability, deterioration, or rehabilitation need. Decisions require information about function, residence, caregivers, family support, and patient preferences.

These findings may motivate research on discharge support, but no prediction rule or intervention was validated. They do not establish that using age, CCI, or physiological measurements for discharge decisions improves outcomes.

### 4.4. Limitations

First, the retrospective design cannot establish causality. Unmeasured frailty, activities of daily living, mobility, cognition, residence, caregiver availability, pain treatment, and rehabilitation may confound associations. CCI incompleteness and component-level no-point coding may misclassify baseline health.

Second, registry participation may underrepresent minor injuries treated elsewhere. Stringent coded eligibility and survivor restriction limit generalizability. Conditioning on survival may distort associations if survival shares determinants with patient characteristics or injury severity; the bias direction cannot be established here.

Third, a common AIS code does not ensure uniform injury burden. Exact fracture counts above three and unrecorded injuries were unavailable. Expanded-eligibility findings differed for some associations, reinforcing population dependence rather than uniform robustness.

Fourth, complete-case selection and exclusion of unavailable discharge destinations may bias estimates. Descriptive comparisons do not establish random missingness. The larger cohort excludes 84 other/unavailable destinations and one additional confirmed home discharge with missing CCI. Bias direction remains uncertain.

Fifth, hospital identifiers were not provided. We could not adjust for within-hospital dependence or practices; CIs may be too narrow and institutional differences may confound associations. HC1 covariance and year adjustment do not resolve clustering or institution-specific pandemic effects.

Sixth, recorded LOS has limited temporal resolution; partial-day handling was not independently verified. Zero days, long stays, and distributional assumptions influenced some findings. Excluding zeros changes the population and does not validate an ordinary untruncated likelihood for positive stays.

Seventh, NB models improved on Poisson dispersion but retained lack of fit. Flexible-age and categorical-GCS results limit constant-effect interpretations. Sparse events restrict smaller-cohort transfer precision; Firth correction and single-deletion stability do not remove this uncertainty.

Finally, models and added sensitivity analyses were developed during analysis and are exploratory. Multiple testing without adjustment permits chance findings. Separate group *p*-values do not establish between-group differences. Added analyses assess specific concerns rather than independently validate the primary findings.

## 5. Conclusions

Among selected adult survivors with target rib-fracture codes and restricted concomitant injuries, higher CCI was associated with greater odds of interhospital transfer. Lower GCS was associated with longer modeled mean LOS in the three-or-more-rib group. Some estimates depended on eligibility or model specification, and age associations were not uniformly linear. Selection, incomplete information, and unmeasured functional and institutional characteristics limit interpretation.

The study provides incremental evidence on hospital course and disposition in this registry-defined population. It does not establish causal effects, validated individual prediction, or benefit from a discharge-management strategy.

## Figures and Tables

**Table 1:** Patient characteristics and outcomes.

Variable	1–2-Rib Fracture Group (n = 149)	Obs./ Miss.	≥3-Rib Fracture Group (n = 787)	Obs./ Miss.
Number of fractured ribs		149/0		787/0
1 rib	74 (49.7%)		—	
2 ribs	75 (50.3%)		—	
≥3 ribs	—		787 (100.0%)	
Age, years	60.5 ± 19.7	149/0	68.0 ± 16.7	787/0
Year of admission		149/0		787/0
2019	60 (40.3%)		253 (32.1%)	
2020	53 (35.6%)		300 (38.1%)	
2021	36 (24.2%)		234 (29.7%)	
Sex		148/1		761/26
Male	96 (64.9%)		440 (57.8%)	
Female	52 (35.1%)		321 (42.2%)	
Injury mechanism		149/0		787/0
Traffic injury	82 (55.0%)		346 (44.0%)	
Falls	50 (33.6%)		369 (46.9%)	
Other/unclassifiable	17 (11.4%)		72 (9.1%)	
CCI, points	0.5 ± 0.9	149/0	0.6 ± 1.2	786/1
Arrival systolic BP, mmHg	148.4 ± 29.9	148/1	149.2 ± 28.6	780/7
Arrival diastolic BP, mmHg	87.9 ± 18.6	148/1	86.7 ± 17.9	747/40
Arrival heart rate, beats/min	84.1 ± 15.6	147/2	82.8 ± 16.6	751/36
Arrival respiratory rate, breaths/min	19.7 ± 4.7	140/9	20.6 ± 5.4	752/35
Arrival GCS, points	14.5 ± 1.6	135/14	14.6 ± 1.0	765/22
Arrival SpO_2_, %	97.9 ± 2.4	133/16	97.2 ± 2.9	708/79
Oxygen administration		132/17		656/131
Yes	61 (46.2%)		249 (38.0%)	
No	71 (53.8%)		407 (62.0%)	
LOS, days	5.1 ± 9.7	140/9	7.5 ± 17.3	744/43
Discharge disposition		144/5		716/71
Home	119 (82.6%)		545 (76.1%)	
Another medical institution	23 (16.0%)		158 (22.1%)	
Other	2 (1.4%)		13 (1.8%)	

Continuous variables are mean ± SD; categorical variables are n (%), with nonmissing denominators. Obs./miss. means observed/missing or invalid. Unknown physiological flags take precedence over numeric values; RR ≥60 is unavailable. Zero-day LOS is retained. Disposition other than home/transfer is shown descriptively but excluded from transfer models. CCI, Charlson Comorbidity Index; GCS, Glasgow Coma Scale; BP, blood pressure; LOS, recorded index-hospital length of stay.

**Table 2:** Factors associated with LOS.

Variable and Contrast/Unit	1–2-Rib Fracture Group (n = 126)	*p*-Value	≥3-Rib Fracture Group (n = 694)	*p*-Value
Age Per 10 years	1.166 (1.013–1.343)	0.032	1.231 (1.156–1.310)	6.87 × 10^−11^
Injury mechanism Traffic injury/falls	0.535 (0.313–0.912)	0.022	0.856 (0.700–1.047)	0.130
Injury mechanism Traffic injury/other or unclassifiable	0.425 (0.213–0.849)	0.015	0.897 (0.592–1.358)	0.607
Fracture number 2 ribs/1 rib	1.936 (1.227–3.055)	0.005	—	—
CCI Per point	1.042 (0.776–1.399)	0.785	0.982 (0.904–1.067)	0.663
Arrival systolic BP Per 10 mmHg	1.107 (1.019–1.203)	0.017	0.982 (0.950–1.015)	0.292
Arrival respiratory rate Per 5 breaths/min	0.999 (0.791–1.264)	0.996	1.003 (0.921–1.092)	0.945
Arrival GCS Per point	1.075 (0.931–1.241)	0.325	0.796 (0.722–0.879)	6.12 × 10^−6^

Adjusted mean LOS ratios (95% CIs), NB2 log-link complete-case models; covariates are all listed variables (fracture number in 1–2 ribs only). Zero-day counts: 19/95. Missing/invalid LOS: 9/43; further covariate exclusions: 14/50. CIs and *p*-values use model-based Wald inference. Traffic contrasts derive from one model. Age ratios are linear summaries; see Figure S1. Numerical *p*-values are not multiplicity-adjusted.

**Table 3:** Factors associated with interhospital transfer.

Variable and Contrast/Unit	1–2-Rib Fracture Group (n = 142)	*p*-Value	≥3-rib Fracture Group (n = 702)	*p*-Value
Age Per 10 years	1.476 (1.061–2.139)	0.020	1.353 (1.185–1.554)	4.21 × 10^−6^
Injury mechanism Traffic injury/falls	1.176 (0.352–4.236)	0.795	0.797 (0.532–1.192)	0.270
Injury mechanism Traffic injury/other or unclassifiable	0.376 (0.088–1.838)	0.213	0.503 (0.241–1.101)	0.084
CCI Per point	2.630 (1.531–4.960)	2.94 × 10^−4^	1.278 (1.109–1.479)	6.66 × 10^−4^

Values are adjusted transfer odds ratios (95% CIs) from Firth logistic models with age, mechanism, and CCI; home discharge is the reference outcome. Events are 23/142 and 158/702. One missing CCI is excluded in the larger cohort. CIs use penalized profile likelihood; *p*-values use two-sided penalized likelihood-ratio tests. Age ratios are linear summaries; larger-cohort age nonlinearity is shown in Figure S1 and Table S14. Abbreviations follow Table 1. Traffic-injury contrasts derive from the same model with changed reference categories as needed. Numerical *p*-values use three decimals or scientific notation below 0.001. They are not multiplicity-adjusted. Odds ratios do not express proportional changes in transfer probability.

## Data Availability

Patient-level JTDB data are restricted and cannot be distributed by the authors. Access requires compliance with registry regulations and approval procedures. The supplied reproducibility package contains the cohort-selection algorithm, variable definitions, and statistical code without patient records, identifiers, or patient-level outputs. Reproducing numerical results requires authorized access to the underlying data.

## Supplementary Materials

The following supporting information can be downloaded at: https://www.mdpi.com/article/10.3390/jcm15218728/s1, Figure S1: Flexible age associations relative to age 60; Table S1: Unadjusted comparisons by discharge disposition in the 1–2-rib fracture group; Table S2: Unadjusted comparisons by discharge disposition in the ≥3-rib fracture group; Table S3: Excluding the longest LOS in each group; Table S4: LOS with expanded eligibility; Table S5: Transfer with expanded eligibility; Table S6: Accounting for complete-case LOS exclusions; Table S7: Included and excluded patients in complete-case LOS analyses; Table S8: Initial LOS model diagnostics; Table S9: Excluding zero-day records;. Table S10: Excluding LOS above the within-group 99th percentile; Table S11: Alternative Poisson mean model with HC1 covariance; Table S12: Categorical GCS sensitivity assessment; Table S13: Assessments of continuous-variable nonlinearity; Table S14: Age-specific contrasts from spline sensitivity models; Table S15: Components of the mechanism classification; Table S16: Separating same-level falls in the larger cohort; Table S17: Excluding unavailable mechanism information; Table S18: Calendar-year adjustment; Table S19: Leave-one-out assessment of the small transfer model; Table S20: Sequential eligibility and coding algorithm.
