# Supplementary material for: Factors Associated with Hospital Length of Stay and Interhospital Transfer in Patients with Rib Fractures: A Retrospective Study Using the Japan Trauma Data Bank
Source: J Clin Med. 2026 Nov 9;15(21):8728. doi: 10.3390/jcm15218728 (PMC13764552; doi:10.3390/jcm15218728)
Supplement: Supplementary file 1 [file jcm-15-08728-s001.zip › jcm-4644773-supplementary.pdf]

Supplementary analyses of recorded LOS and interhospital transfer

Primary eligibility: target rib-fracture AIS codes, no additional recorded chest AIS codes, adult survivors without recorded chest compressions, and maximum extra-chest AIS <2. The RR ≥60 is unavailable. Tables use the updated complete-case datasets. p-values are numerical and not multiplicity-adjusted. The expanded-eligibility analyses permit maximum extra-chest AIS ≤2.

Supplementary Table S1. Unadjusted comparisons by discharge disposition in the 1–2-rib fracture group

Variable	Home (n=119)	Obs. n	Transfer (n=23)	Obs. n	p-value
Number of fractured ribs					
1-rib fracture	64 (53.8%)	119	8 (34.8%)	23	0.114
2-rib fracture	55 (46.2%)	119	15 (65.2%)	23	
Age, years	57.0 ± 19.2	119	75.7 ± 16.2	23	2.22×10^{-5}
Injury mechanism					
Traffic injury	71 (59.7%)	119	8 (34.8%)	23	0.057
Falls	34 (28.6%)	119	12 (52.2%)	23	
Other/unclassifiable	14 (11.8%)	119	3 (13.0%)	23	
CCI, points	0.3 ± 0.6	119	1.4 ± 1.2	23	2.52×10^{-4}
Arrival systolic BP, mmHg	149.5 ± 27.8	118	146.3 ± 35.7	23	0.689
Arrival diastolic BP, mmHg	89.3 ± 17.7	118	83.6 ± 16.1	23	0.134
Arrival heart rate, beats/min	83.4 ± 15.0	118	89.6 ± 16.3	22	0.110
Arrival respiratory rate, breaths/min	19.9 ± 4.9	111	18.8 ± 4.0	22	0.242
Arrival GCS, points	14.5 ± 1.6	106	14.6 ± 0.7	22	0.597
Arrival SpO_2, %	98.1 ± 2.2	106	96.9 ± 3.6	20	0.151
Oxygen administration					
Yes	51 (47.7%)	107	6 (33.3%)	18	0.312
No	56 (52.3%)	107	12 (66.7%)	18	

Continuous variables are mean ± SD; categorical variables are n (%) using each variable's nonmissing denominator. Obs. n is the number available for that variable. Welch t-tests compare continuous variables; Fisher exact tests compare categorical variables, with one overall p-value per factor. Physiological unknown flags take precedence over numeric values. No missing-data imputation was performed. Falls combines same-level falls and other recorded fall categories. BP, blood pressure; CCI, Charlson Comorbidity Index; GCS, Glasgow Coma Scale; SpO_2, peripheral oxygen saturation.

Supplementary Table S2. Unadjusted comparisons by discharge disposition in the ≥3-rib fracture group

Variable	Home (n=545)	Obs. n	Transfer (n=158)	Obs. n	p-value
Age, years	66.2 ± 16.5	545	75.1 ± 15.8	158	2.98×10^{-9}
Injury mechanism					
Traffic injury	271 (49.7%)	545	55 (34.8%)	158	0.003
Falls	245 (45.0%)	545	91 (57.6%)	158	
Other/unclassifiable	29 (5.3%)	545	12 (7.6%)	158	
CCI, points	0.5 ± 1.0	544	1.1 ± 1.5	158	3.51×10^{-5}
Arrival systolic BP, mmHg	149.1 ± 28.2	539	151.8 ± 28.4	158	0.289
Arrival diastolic BP, mmHg	86.7 ± 18.0	535	85.9 ± 17.5	158	0.630
Arrival heart rate, beats/min	82.7 ± 16.2	538	83.4 ± 18.5	158	0.672
Arrival respiratory rate, breaths/min	20.4 ± 5.3	523	20.7 ± 5.7	149	0.478
Arrival GCS, points	14.7 ± 0.7	528	14.3 ± 1.4	153	4.92×10^{-4}
Arrival SpO_2, %	97.4 ± 2.7	513	96.6 ± 3.5	147	0.017
Oxygen administration					
Yes	181 (38.0%)	476	53 (38.7%)	137	0.921
No	295 (62.0%)	476	84 (61.3%)	137	

Continuous variables are mean ± SD; categorical variables are n (%) using each variable's nonmissing denominator. Obs. n is the number available for that variable. Welch t-tests compare continuous variables; Fisher exact tests compare categorical variables, with one overall p-value per factor. Physiological unknown flags take precedence over numeric values. No missing-data imputation was performed. Falls combines same-level falls and other recorded fall categories. BP, blood pressure; CCI, Charlson Comorbidity Index; GCS, Glasgow Coma Scale; SpO_2, peripheral oxygen saturation.

Supplementary Table S3. Excluding the longest LOS in each group

Variable and contrast/unit	1–2-rib fracture group (n=125)	p-value	≥3-rib fracture group (n=693)	p-value
Age<br>Per 10 years	1.092<br>(0.955–1.248)	0.199	1.233<br>(1.161–1.309)	8.50×10^{-12}
Injury mechanism<br>Traffic injury / falls	0.592<br>(0.355–0.986)	0.044	0.986<br>(0.813–1.196)	0.884
Injury mechanism<br>Traffic injury / other or unclassifiable	0.458<br>(0.238–0.883)	0.020	0.886<br>(0.595–1.319)	0.551
Fracture number<br>2-rib fracture / 1-rib fracture	2.476<br>(1.597–3.839)	5.11×10^{-5}	—	—
CCI<br>Per point	1.211<br>(0.917–1.600)	0.177	1.026<br>(0.948–1.111)	0.523
Arrival systolic BP<br>Per 10 mmHg	1.051<br>(0.971–1.138)	0.219	0.995<br>(0.964–1.028)	0.777
Arrival respiratory rate<br>Per 5 breaths/min	1.106<br>(0.885–1.383)	0.375	1.015<br>(0.936–1.102)	0.714
Arrival GCS<br>Per point	1.024<br>(0.898–1.169)	0.719	0.847<br>(0.770–0.931)	5.93×10^{-4}

Values are adjusted mean LOS ratios from NB2 log-link regression; 95% CIs and two-sided p-values use model-based Wald inference.

Covariates are age, mechanism, CCI, SBP, RR, GCS, and fracture number in the smaller cohort. Models use these covariates unless the table specifies a modification. Age ratios are summaries under a linear-age specification, not proof of a constant association across ages. CCI, Charlson Comorbidity Index; BP, blood pressure; RR, respiratory rate; GCS, Glasgow Coma Scale; LOS, recorded index-hospital length of stay. Analyses are exploratory, without multiplicity adjustment.

The recorded maxima of 89 and 381 days were excluded, leaving 125 and 693 complete cases in each group. These source values were not independently validated against clinical charts.

Supplementary Table S4. LOS with expanded eligibility

Variable and contrast/unit	1–2-rib fracture group (n=566)	p-value	≥3-rib fracture group (n=1889)	p-value
Age<br>Per 10 years	1.036<br>(0.980–1.094)	0.215	1.135<br>(1.097–1.175)	5.06×10^{-13}
Injury mechanism<br>Traffic injury / falls	0.711<br>(0.581–0.870)	9.10×10^{-4}	1.061<br>(0.952–1.183)	0.282
Injury mechanism<br>Traffic injury / other or unclassifiable	1.370<br>(0.931–2.015)	0.110	0.689<br>(0.550–0.864)	0.001
Fracture number<br>2-rib fracture / 1-rib fracture	0.967<br>(0.805–1.162)	0.721	—	—
CCI<br>Per point	0.980<br>(0.891–1.079)	0.686	0.967<br>(0.918–1.019)	0.208
Arrival systolic BP<br>Per 10 mmHg	1.011<br>(0.977–1.046)	0.517	0.964<br>(0.947–0.981)	5.41×10^{-5}
Arrival respiratory rate<br>Per 5 breaths/min	1.070<br>(0.975–1.175)	0.153	1.022<br>(0.973–1.072)	0.388
Arrival GCS<br>Per point	0.938<br>(0.885–0.995)	0.032	0.918<br>(0.879–0.959)	1.14×10^{-4}

Values are adjusted mean LOS ratios from NB2 log-link regression; 95% CIs and two-sided p-values use model-based Wald inference.

Covariates are age, mechanism, CCI, SBP, RR, GCS, and fracture number in the smaller cohort. Models use these covariates unless the table specifies a modification. Age ratios are summaries under a linear-age specification, not proof of a constant association across ages. CCI, Charlson Comorbidity Index; BP, blood pressure; RR, respiratory rate; GCS, Glasgow Coma Scale; LOS, recorded index-hospital length of stay. Analyses are exploratory, without multiplicity adjustment.

Expanded eligible samples are 632 and 2,091 in each group; complete-case LOS samples are 566 and 1,889 in each. All other eligibility criteria are retained. Negative LOS values are unavailable, including a −19-day record among additional smaller-cohort patients.

Supplementary Table S5. Transfer with expanded eligibility

Variable and contrast/unit	1–2-rib fracture group (n=599)	p-value	≥3-rib fracture group (n=1954)	p-value
Age<br>Per 10 years	1.289<br>(1.164–1.433)	6.82×10^{-7}	1.387<br>(1.296–1.485)	5.67×10^{-23}
Injury mechanism<br>Traffic injury / falls	0.958<br>(0.660–1.394)	0.820	0.961<br>(0.785–1.177)	0.699
Injury mechanism<br>Traffic injury / other or unclassifiable	0.963<br>(0.456–2.164)	0.923	0.697<br>(0.458–1.069)	0.097
CCI<br>Per point	1.137<br>(0.963–1.357)	0.131	1.127<br>(1.026–1.239)	0.012

Values are adjusted transfer odds ratios from Firth logistic regression; 95% CIs use penalized profile likelihood and two-sided p-values use penalized likelihood-ratio tests. Home discharge is the reference outcome.

Covariates are age, mechanism, and CCI. Models use these covariates unless the table specifies a modification. Age ratios are summaries under a linear-age specification, not proof of a constant association across ages. CCI, Charlson Comorbidity Index; BP, blood pressure; RR, respiratory rate; GCS, Glasgow Coma Scale; LOS, recorded index-hospital length of stay. Analyses are exploratory, without multiplicity adjustment.

Expanded transfer samples are 599 (210 events) and 1,954 (710 events) in each group. RR is not a transfer covariate.

Supplementary Table S6. Accounting for complete-case LOS exclusions

**Selection or missing variable**	**1–2-rib fracture**	**≥3-rib fracture**
Eligible	149	787
Unavailable/invalid LOS	9	43
Valid recorded LOS	140	744
Missing ≥1 covariate among valid LOS	14	50
SBP unavailable	1	7
RR unavailable	9	32
GCS unavailable	8	20
Complete-case LOS	126	694
Excluded from LOS model overall	23 (15.4%)	93 (11.8%)
Zero-day LOS in eligible cohort	20	114
Zero-day LOS in complete cases	19	95

Variable-specific missing counts overlap. Among valid-LOS patients, age, CCI, mechanism coding, and the applicable fracture category were available. RR cleaning reduced the larger complete-case sample from 696 to 694 in ≥3-rib fracture group.

Supplementary Table S7. Included and excluded patients in complete-case LOS analyses

Variable	1–2-rib fracture Included n=126	1–2-rib fracture Excluded n=23	≥3-rib fracture Included n=694	≥3-rib fracture Excluded n=93
Age, years	60.6 ± 19.8 (n=126)	60.1 ± 19.9 (n=23)	68.2 ± 16.5 (n=694)	66.3 ± 18.2 (n=93)
CCI, points	0.4 ± 0.8 (n=126)	0.6 ± 1.1 (n=23)	0.6 ± 1.1 (n=694)	0.7 ± 1.4 (n=92)
SBP, mmHg	149.5 ± 28.0 (n=126)	142.3 ± 39.3 (n=22)	150.0 ± 28.5 (n=694)	143.1 ± 28.0 (n=86)
RR, breaths/min	19.5 ± 4.8 (n=126)	21.4 ± 4.0 (n=14)	20.5 ± 5.4 (n=694)	21.4 ± 5.9 (n=58)
GCS, points	14.5 ± 1.5 (n=126)	13.9 ± 3.0 (n=9)	14.7 ± 0.9 (n=694)	14.5 ± 1.4 (n=71)
LOS, days	5.3 ± 10.2 (n=126)	3.7 ± 3.9 (n=14)	7.7 ± 17.7 (n=694)	5.6 ± 10.1 (n=50)
Male	79 (63.2%) (n=125)	17 (73.9%) (n=23)	400 (57.9%) (n=691)	40 (57.1%) (n=70)
Traffic injury	67 (53.2%) (n=126)	15 (65.2%) (n=23)	319 (46.0%) (n=694)	27 (29.0%) (n=93)
Falls	44 (34.9%) (n=126)	6 (26.1%) (n=23)	337 (48.6%) (n=694)	32 (34.4%) (n=93)
Other/unclassifiable	15 (11.9%) (n=126)	2 (8.7%) (n=23)	38 (5.5%) (n=694)	34 (36.6%) (n=93)
Transfer among confirmed home/transfer	21 (17.2%) (n=122)	2 (10.0%) (n=20)	145 (22.4%) (n=646)	13 (22.8%) (n=57)

Comparisons are descriptive. Each cell uses available observations: excluded-patient LOS describes only 14 and 50 patients with valid LOS. Included/excluded refers to the LOS model, not transfer-model eligibility. These comparisons do not establish random missingness or identify the direction of adjusted-association bias.

Supplementary Table S8. Initial LOS model diagnostics

**Diagnostic**	**1–2-rib fracture**	**≥3-rib fracture**
n	126	694
Mean LOS	5.294	7.679
Variance of LOS	103.137	312.545
Observed zeros	19	95
Expected zeros under NB	30.548	125.916
NB theta	0.862	0.744
NB alpha	1.160	1.345
Poisson Pearson χ^2/df	11.079	34.107
NB Pearson χ^2/df	1.383	2.675
Poisson AIC	1229.044	10260.840
NB AIC	661.093	4182.779

NB2 variance is $\mu+\alpha\mu^2$; theta=1/α. Both models converged. Better dispersion and AIC than Poisson do not establish adequate fit throughout the distribution. Expected zero counts exceed observed counts. These diagnostics do not account for institutional clustering.

Supplementary Table S9. Excluding zero-day records

Variable and contrast/unit	1–2-rib fracture group (n=107)	p-value	≥3-rib fracture group (n=599)	p-value
Age<br>Per 10 years	1.120<br>(0.983–1.277)	0.089	1.204<br>(1.138–1.273)	1.15×10^{-10}
Injury mechanism<br>Traffic injury / falls	0.580<br>(0.353–0.953)	0.031	0.812<br>(0.678–0.971)	0.023
Injury mechanism<br>Traffic injury / other or unclassifiable	0.518<br>(0.270–0.994)	0.048	0.875<br>(0.602–1.270)	0.482
Fracture number<br>2-rib fracture / 1-rib fracture	1.579<br>(1.024–2.435)	0.039	—	—
CCI<br>Per point	0.997<br>(0.766–1.298)	0.980	0.972<br>(0.904–1.044)	0.435
Arrival systolic BP<br>Per 10 mmHg	1.119<br>(1.033–1.211)	0.006	0.985<br>(0.956–1.015)	0.318
Arrival respiratory rate<br>Per 5 breaths/min	1.008<br>(0.806–1.261)	0.942	0.970<br>(0.900–1.046)	0.430
Arrival GCS<br>Per point	1.030<br>(0.872–1.217)	0.728	0.794<br>(0.727–0.868)	3.36×10^{-7}

Values are adjusted mean LOS ratios from NB2 log-link regression; 95% CIs and two-sided p-values use model-based Wald inference.

Covariates are age, mechanism, CCI, SBP, RR, GCS, and fracture number in the smaller cohort. Models use these covariates unless the table specifies a modification. Age ratios are summaries under a linear-age specification, not proof of a constant association across ages. CCI, Charlson Comorbidity Index; BP, blood pressure; RR, respiratory rate; GCS, Glasgow Coma Scale; LOS, recorded index-hospital length of stay. Analyses are exploratory, without multiplicity adjustment.

The 19 and 95 zero-day records were excluded in each group. This restricts the population to positive recorded LOS. Refitting an ordinary NB model to this subset is a descriptive sensitivity assessment, not a zero-truncated likelihood analysis or a validation of distributional assumptions.

Supplementary Table S10. Excluding LOS above the within-group 99th percentile

Variable and contrast/unit	1–2-rib fracture group (n=124)	p-value	≥3-rib fracture group (n=687)	p-value
Age<br>Per 10 years	1.116<br>(0.980–1.270)	0.098	1.193<br>(1.126–1.264)	2.43×10^{-9}
Injury mechanism<br>Traffic injury / falls	0.481<br>(0.294–0.788)	0.004	0.969<br>(0.804–1.167)	0.739
Injury mechanism<br>Traffic injury / other or unclassifiable	0.448<br>(0.238–0.844)	0.013	1.011<br>(0.684–1.493)	0.958
Fracture number<br>2-rib fracture / 1-rib fracture	2.273<br>(1.487–3.475)	1.49×10^{-4}	—	—
CCI<br>Per point	1.051<br>(0.798–1.384)	0.722	1.031<br>(0.955–1.112)	0.437
Arrival systolic BP<br>Per 10 mmHg	0.995<br>(0.920–1.076)	0.891	0.988<br>(0.959–1.019)	0.460
Arrival respiratory rate<br>Per 5 breaths/min	1.144<br>(0.922–1.419)	0.221	1.036<br>(0.958–1.121)	0.377
Arrival GCS<br>Per point	1.044<br>(0.918–1.187)	0.508	0.848<br>(0.775–0.929)	4.01×10^{-4}

Values are adjusted mean LOS ratios from NB2 log-link regression; 95% CIs and two-sided p-values use model-based Wald inference.

Covariates are age, mechanism, CCI, SBP, RR, GCS, and fracture number in the smaller cohort. Models use these covariates unless the table specifies a modification. Age ratios are summaries under a linear-age specification, not proof of a constant association across ages. CCI, Charlson Comorbidity Index; BP, blood pressure; RR, respiratory rate; GCS, Glasgow Coma Scale; LOS, recorded index-hospital length of stay. Analyses are exploratory, without multiplicity adjustment.

Type-7 sample 99th percentiles are 43.50 and 50.14 days of hospital stay, excluding 2 and 7 patients in each group. The thresholds were selected for this exploratory analysis, not prospectively prespecified; excluded records are not presumed erroneous.

Supplementary Table S11. Alternative Poisson mean model with HC1 covariance

Variable and contrast/unit	1–2-rib fracture group (n=126)	p-value	≥3-rib fracture group (n=694)	p-value
Age<br>Per 10 years	1.224<br>(0.964–1.555)	0.097	1.235<br>(1.144–1.333)	5.87×10^{-8}
Injury mechanism<br>Traffic injury / falls	0.560<br>(0.299–1.049)	0.070	0.820<br>(0.579–1.160)	0.262
Injury mechanism<br>Traffic injury / other or unclassifiable	0.737<br>(0.242–2.245)	0.592	0.842<br>(0.485–1.460)	0.540
Fracture number<br>2-rib fracture / 1-rib fracture	1.418<br>(0.662–3.037)	0.368	—	—
CCI<br>Per point	0.975<br>(0.618–1.539)	0.914	0.990<br>(0.893–1.099)	0.854
Arrival systolic BP<br>Per 10 mmHg	1.090<br>(0.967–1.230)	0.159	0.988<br>(0.952–1.026)	0.544
Arrival respiratory rate<br>Per 5 breaths/min	0.929<br>(0.686–1.260)	0.637	1.015<br>(0.939–1.097)	0.702
Arrival GCS<br>Per point	1.038<br>(0.932–1.155)	0.499	0.850<br>(0.802–0.902)	6.69×10^{-8}

Values are adjusted mean LOS ratios from a Poisson log-link mean model; 95% CIs and two-sided p-values use HC1 sandwich covariance.

Covariates are age, mechanism, CCI, SBP, RR, GCS, and fracture number in the smaller cohort. Models use these covariates unless the table specifies a modification. Age ratios are summaries under a linear-age specification, not proof of a constant association across ages. CCI, Charlson Comorbidity Index; BP, blood pressure; RR, respiratory rate; GCS, Glasgow Coma Scale; LOS, recorded index-hospital length of stay. Analyses are exploratory, without multiplicity adjustment.

Poisson coefficients are estimated by log-link score equations. CIs and p-values use a heteroskedasticity-consistent sandwich covariance with $n/(n-p)$ HC1 correction, not Poisson model-based variance. This does not address mean-model misspecification or hospital clustering.

Supplementary Table S12. Categorical GCS sensitivity assessment

Group	n	GCS <15 / 15<br>Adjusted mean LOS ratio (95% CI)	p-value
1–2-rib fracture	126	1.048<br>(0.617–1.778)	0.863
≥3-rib fracture	694	1.924<br>(1.542–2.400)	6.81×10^{-9}

The GCS of 15 occurs in 101/126 and 547/694, primary complete cases in each group. Scores below 14 occur in 8 and 43 in each group. NB2 log-link models replace continuous GCS with the <15 indicator and retain age, mechanism, CCI, SBP, RR, and smaller-cohort fracture number. CIs and p-values use model-based Wald inference. The threshold is exploratory, not validated. Categorization does not prove a constant per-point effect.

Supplementary Table S13. Assessments of continuous-variable nonlinearity

Model	Variable	Knots at 10th/50th/90th percentiles	Nonlinearity p
1–2-rib fracture LOS	Age, years	31.5 / 61 / 86.5	0.004
1–2-rib fracture LOS	SBP, mmHg	115 / 147.5 / 185	0.706
1–2-rib fracture LOS	RR, breaths/min	14 / 19 / 25.5	0.588
≥3-rib fracture LOS	Age, years	45 / 71 / 88	0.809
≥3-rib fracture LOS	SBP, mmHg	114 / 149 / 185	0.207
≥3-rib fracture LOS	RR, breaths/min	15 / 20 / 27	0.616
≥3-rib fracture Transfer	Age, years	44 / 72 / 88	0.004

A three-knot restricted cubic spline adds one nonlinear degree of freedom while retaining the linear term. LOS tests compare NB models by likelihood ratio; transfer uses a penalized likelihood-ratio test within the full Firth penalty. Tests are exploratory. GCS percentile knots coincide at 15, so its distribution and categorical sensitivity are reported instead. The small transfer cohort was not expanded with a nonlinear age term because of its 23 events. Nonsignificance does not establish linearity.

Supplementary Table S14. Age-specific contrasts from spline sensitivity models

Model	n	Age contrast	Adjusted ratio (95% CI)
1–2-rib fracture LOS	126	40 / 60 years	1.008 (0.711–1.429)
1–2-rib fracture LOS	126	80 / 60 years	2.171 (1.439–3.277)
≥3-rib fracture LOS	694	40 / 60 years	0.675 (0.540–0.842)
≥3-rib fracture LOS	694	80 / 60 years	1.529 (1.323–1.768)
≥3-rib fracture Transfer	702	40 / 60 years	1.045 (0.654–1.671)
≥3-rib fracture Transfer	702	80 / 60 years	2.061 (1.579–2.692)

Ratios compare the modeled mean recorded LOS or odds of transfer at the specified age with age 60, conditional on other included covariates. These are spline-model contrasts, not per-10-year effects. Pointwise CIs here use a Wald delta method for both outcomes (distinct from the profile CIs in primary Firth tables). They are not simultaneous bands. No multiplicity adjustment was applied.

Supplementary Figure S1. Flexible age associations relative to age 60

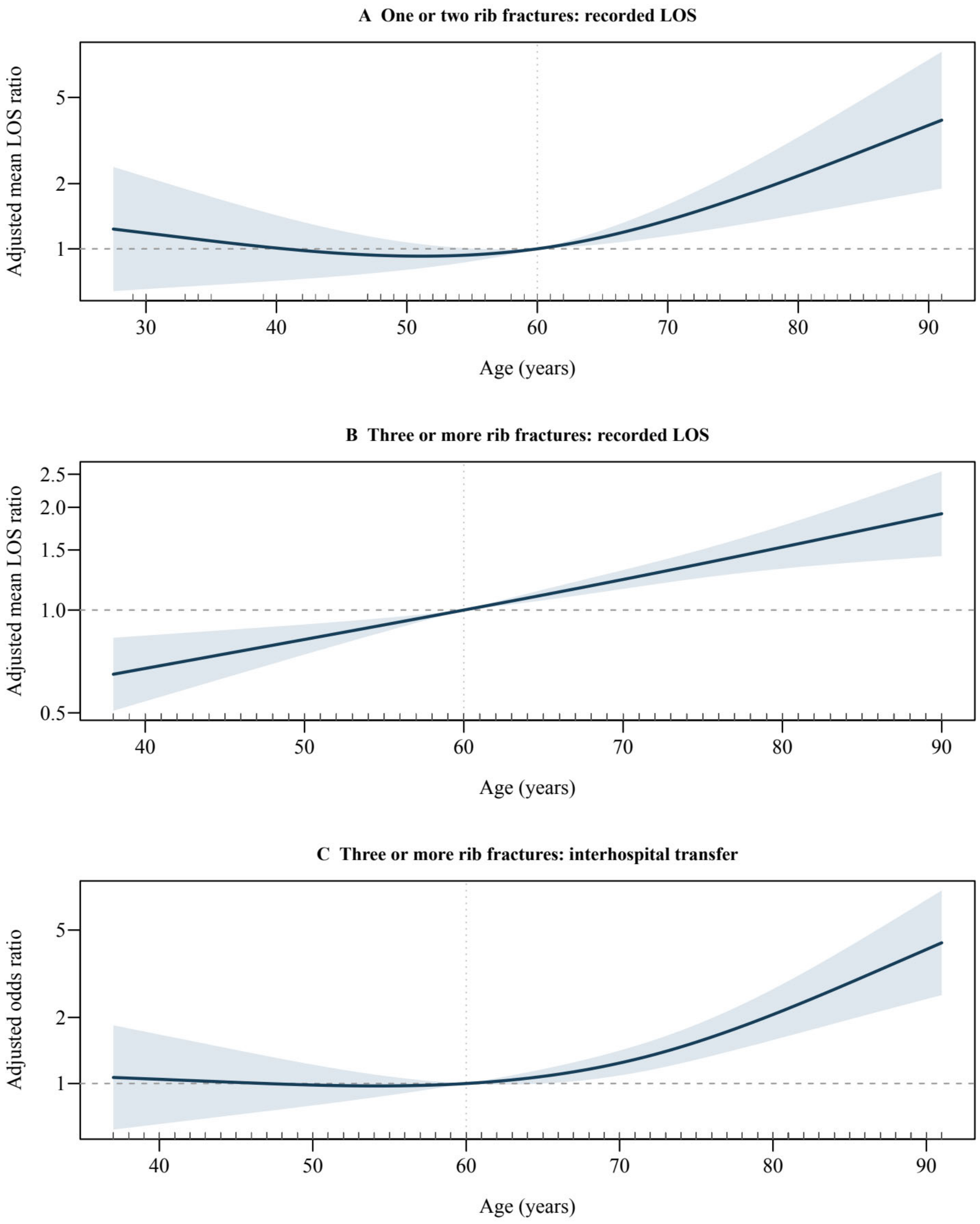

Three-knot restricted cubic splines use Table S13 knots. Lines show adjusted ratios relative to age 60; shaded regions are pointwise Wald 95% CIs. The vertical scale is logarithmic. Rug marks indicate observed ages; curves display the central 5th–95th percentile range. The reference-age CI collapses to 1 by definition. A: 1–2-rib fracture LOS; B: ≥3-rib fracture LOS; C: ≥3-rib fracture transfer. Curves are exploratory and do not represent causal effects or validated individual predictions.

Supplementary Table S15. Components of the mechanism classification

Recorded mechanism	1–2-rib fracture n=149	≥3-rib fracture n=787
Traffic injury	82	346
Same-level fall (tento)	27	211
Fall such as stairs (tenraku)	19	126
Separate fall category (tsuiraku)	4	32
Recorded other mechanism	12	38
No mechanism recorded	5	34

The source labels are transliterated as tento, tenraku, and tsuiraku. They distinguish fall categories but do not supply precise heights; no height threshold is inferred. Known other mechanisms include interpersonal/object forces, machinery, crush, and other recorded events; no-record cases are distinct from these. Eligible-population counts differ from model complete cases.

Supplementary Table S16. Separating same-level falls in the larger cohort

Group and outcome	n	Variable or contrast	Adjusted ratio (95% CI)	p-value
≥3-rib fracture LOS	694	Age Per 10 years	1.238 (1.161–1.319)	5.25×10^{-11}
≥3-rib fracture LOS	694	CCI Per point	0.986 (0.907–1.071)	0.734
≥3-rib fracture LOS	694	Arrival systolic BP Per 10 mmHg	0.983 (0.951–1.016)	0.305
≥3-rib fracture LOS	694	Arrival respiratory rate Per 5 breaths/min	0.997 (0.916–1.086)	0.950
≥3-rib fracture LOS	694	Arrival GCS Per point	0.798 (0.723–0.881)	7.52×10^{-6}
≥3-rib fracture LOS	694	Traffic injury / same-level fall	0.906 (0.712–1.152)	0.419
≥3-rib fracture LOS	694	Traffic injury / other fall	0.812 (0.636–1.036)	0.094
≥3-rib fracture LOS	694	Injury mechanism Traffic injury / other or unclassifiable	0.902 (0.595–1.365)	0.625
≥3-rib fracture Transfer	702	Age Per 10 years	1.312 (1.147–1.510)	5.24×10^{-5}
≥3-rib fracture Transfer	702	CCI Per point	1.264 (1.096–1.465)	0.001
≥3-rib fracture Transfer	702	Traffic injury / same-level fall	0.623 (0.394–0.986)	0.043
≥3-rib fracture Transfer	702	Traffic injury / other fall	1.104 (0.661–1.883)	0.709
≥3-rib fracture Transfer	702	Injury mechanism Traffic injury / other or unclassifiable	0.503 (0.242–1.093)	0.081

LOS entries are mean LOS ratios; Transfer entries are odds ratios. LOS covariates are age, mechanism, CCI, SBP, RR, GCS, plus fracture number in the smaller cohort; transfer covariates are age, mechanism, and CCI. Year adjustment adds year indicators. Model-specific inference follows Tables 2–3. Age remains linear here to isolate the coding/year change; nonlinearity is assessed separately. No multiplicity adjustment was applied.

Same-level falls are separated from the combined other fall categories. No additional subdivision was fitted in the small transfer cohort: only 2 events occurred among its non-same-level falls. This does not imply equivalence of mechanisms.

Supplementary Table S17. Excluding unavailable mechanism information

Group and outcome	n	Variable or contrast	Adjusted ratio (95% CI)	p-value
1–2-rib fracture LOS	121	Age Per 10 years	1.197 (1.034–1.386)	0.016
1–2-rib fracture LOS	121	CCI Per point	1.041 (0.776–1.397)	0.790
1–2-rib fracture LOS	121	Arrival systolic BP Per 10 mmHg	1.113 (1.020–1.214)	0.016
1–2-rib fracture LOS	121	Arrival respiratory rate Per 5 breaths/min	0.987 (0.772–1.261)	0.916
1–2-rib fracture LOS	121	Arrival GCS Per point	1.069 (0.928–1.232)	0.357
1–2-rib fracture LOS	121	Fracture number 2-rib fracture / 1-rib fracture	1.906 (1.197–3.034)	0.007
1–2-rib fracture LOS	121	Injury mechanism Traffic injury / falls	0.575 (0.337–0.982)	0.043
1–2-rib fracture LOS	121	Injury mechanism Traffic injury / other or unclassifiable	0.311 (0.141–0.689)	0.004
1–2-rib fracture Transfer	137	Age Per 10 years	1.520 (1.077–2.242)	0.017
1–2-rib fracture Transfer	137	CCI Per point	2.722 (1.571–5.215)	2.08×10^{-4}
1–2-rib fracture Transfer	137	Injury mechanism Traffic injury / falls	1.252 (0.368–4.634)	0.723
1–2-rib fracture Transfer	137	Injury mechanism Traffic injury / other or unclassifiable	0.306 (0.059–1.946)	0.191
≥3-rib fracture LOS	689	Age Per 10 years	1.232 (1.157–1.312)	9.24×10^{-11}
≥3-rib fracture LOS	689	CCI Per point	0.975 (0.897–1.061)	0.560
≥3-rib fracture LOS	689	Arrival systolic BP Per 10 mmHg	0.983 (0.951–1.016)	0.308
≥3-rib fracture LOS	689	Arrival respiratory rate Per 5 breaths/min	1.004 (0.922–1.094)	0.924
≥3-rib fracture LOS	689	Arrival GCS Per point	0.794 (0.719–0.877)	5.06×10^{-6}
≥3-rib fracture LOS	689	Injury mechanism Traffic injury / falls	0.856 (0.700–1.047)	0.131
≥3-rib fracture LOS	689	Injury mechanism Traffic injury / other or unclassifiable	0.951 (0.610–1.484)	0.826
≥3-rib fracture Transfer	697	Age Per 10 years	1.326 (1.161–1.524)	2.05×10^{-5}
≥3-rib fracture Transfer	697	CCI Per point	1.280 (1.111–1.484)	6.50×10^{-4}
≥3-rib fracture Transfer	697	Injury mechanism Traffic injury / falls	0.785 (0.523–1.172)	0.237
≥3-rib fracture Transfer	697	Injury mechanism Traffic injury / other or unclassifiable	0.578 (0.261–1.373)	0.205

LOS entries are mean LOS ratios; Transfer entries are odds ratios. LOS covariates are age, mechanism, CCI, SBP, RR, GCS, plus fracture number in the smaller cohort; transfer covariates are age, mechanism, and CCI. Year adjustment adds year indicators. Model-specific inference follows Tables 2–3. Age remains linear here to isolate the coding/year change; nonlinearity is assessed separately. No multiplicity adjustment was applied.

Model sample sizes are 121/689 for LOS and 137/697 for transfer, with 22/155 events. Although 5/34 eligible patients had no mechanism recorded, only 5 from each cohort entered the primary adjusted models. Exclusion does not establish random missingness.

Supplementary Table S18. Calendar-year adjustment

Group and outcome	n	Variable or contrast	Adjusted ratio (95% CI)	p-value
1–2-rib fracture LOS	126	Age Per 10 years	1.161 (1.010–1.335)	0.036
1–2-rib fracture LOS	126	CCI Per point	1.029 (0.768–1.379)	0.847
1–2-rib fracture LOS	126	Arrival systolic BP Per 10 mmHg	1.094 (1.005–1.190)	0.038
1–2-rib fracture LOS	126	Arrival respiratory rate Per 5 breaths/min	1.021 (0.806–1.294)	0.861
1–2-rib fracture LOS	126	Arrival GCS Per point	1.072 (0.929–1.236)	0.340
1–2-rib fracture LOS	126	Fracture number 2-rib fracture / 1-rib fracture	1.923 (1.223–3.025)	0.005
1–2-rib fracture LOS	126	Injury mechanism Traffic injury / falls	0.528 (0.311–0.899)	0.019
1–2-rib fracture LOS	126	Injury mechanism Traffic injury / other or unclassifiable	0.452 (0.225–0.905)	0.025
1–2-rib fracture LOS	126	2020 / 2019	0.766 (0.455–1.291)	0.317
1–2-rib fracture LOS	126	2021 / 2019	1.170 (0.680–2.013)	0.571
1–2-rib fracture Transfer	142	Age Per 10 years	1.459 (1.051–2.105)	0.023
1–2-rib fracture Transfer	142	CCI Per point	2.584 (1.511–4.862)	3.41×10^{-4}
1–2-rib fracture Transfer	142	Injury mechanism Traffic injury / falls	1.150 (0.348–4.084)	0.821
1–2-rib fracture Transfer	142	Injury mechanism Traffic injury / other or unclassifiable	0.394 (0.093–1.927)	0.234
1–2-rib fracture Transfer	142	2020 / 2019	0.719 (0.212–2.342)	0.583
1–2-rib fracture Transfer	142	2021 / 2019	1.038 (0.274–3.723)	0.955
≥3-rib fracture LOS	694	Age Per 10 years	1.233 (1.159–1.313)	4.44×10^{-11}
≥3-rib fracture LOS	694	CCI Per point	1.000 (0.921–1.085)	0.992
≥3-rib fracture LOS	694	Arrival systolic BP Per 10 mmHg	0.982 (0.950–1.015)	0.290
≥3-rib fracture LOS	694	Arrival respiratory rate Per 5 breaths/min	1.005 (0.924–1.094)	0.906
≥3-rib fracture LOS	694	Arrival GCS Per point	0.817 (0.740–0.902)	6.27×10^{-5}
≥3-rib fracture LOS	694	Injury mechanism Traffic injury / falls	0.858 (0.702–1.047)	0.132
≥3-rib fracture LOS	694	Injury mechanism Traffic injury / other or unclassifiable	0.889 (0.587–1.345)	0.577
≥3-rib fracture LOS	694	2020 / 2019	0.790 (0.633–0.985)	0.036
≥3-rib fracture LOS	694	2021 / 2019	0.741 (0.587–0.937)	0.012
≥3-rib fracture Transfer	702	Age Per 10 years	1.357 (1.188–1.560)	4.01×10^{-6}
≥3-rib fracture Transfer	702	CCI Per point	1.280 (1.111–1.485)	6.42×10^{-4}
≥3-rib fracture Transfer	702	Injury mechanism Traffic injury / falls	0.793 (0.528–1.186)	0.259
≥3-rib fracture Transfer	702	Injury mechanism Traffic injury / other or unclassifiable	0.520 (0.246–1.147)	0.103
≥3-rib fracture Transfer	702	2020 / 2019	0.694 (0.447–1.076)	0.102
≥3-rib fracture Transfer	702	2021 / 2019	0.630 (0.393–1.002)	0.051

LOS entries are mean LOS ratios; Transfer entries are odds ratios. LOS covariates are age, mechanism, CCI, SBP, RR, GCS, plus fracture number in the smaller cohort; transfer covariates are age, mechanism, and CCI. Year adjustment adds year indicators. Model-specific inference follows Tables 2–3. Age remains linear here to isolate the coding/year change; nonlinearity is assessed separately. No multiplicity adjustment was applied.

Year is a categorical covariate, with 2019 as reference; 2020 and 2021 add two coefficients. This adds complexity to the 23-event small transfer model. Calendar year does not measure hospital capacity, restrictions, or institution-specific pandemic effects.

Supplementary Table S19. Leave-one-out assessment of the small transfer model

Variable or contrast	Primary adjusted OR	Range across 142 individual deletions
Age per 10 years	1.476	1.393–1.606
Traffic injury / falls	1.176	0.996–1.633
Traffic injury / other	0.376	0.282–0.582
CCI per point	2.630	2.440–3.396

The same Firth model was refitted after deleting each of the 142 patients; all fits converged. Ranges summarize point estimates, not CIs or validation performance. Nonlinear age was not added to this sparse-event model. Single-deletion stability does not eliminate sparse-data uncertainty or residual confounding.

Supplementary Table S20. Sequential eligibility and coding algorithm

**Selection stage**	**1-rib fracture**	**2-rib fracture**	**≥3-rib fracture**
Target AIS code	450201.1	450202.2	450203.3
Target code in 88,817 registry records	1,889	1,812	11,690
After any other chest AIS code exclusion	1,013	825	3,659
After age <18 / unavailable exclusion	997	816	3,639
After recorded chest compressions exclusion	989	801	3,514
After death exclusion	953	763	3,373
After maximum extra-chest AIS <2	74	75	787
Expanded criterion: maximum extra-chest AIS ≤2	350	282	2,091

Each row applies to the survivors of the preceding shared stage; the final primary and expanded rows are alternative extra-chest criteria after death exclusion. Extra-chest regions are head, face, neck, abdomen, spine, upper limbs, lower limbs, and external. Any additional AIS code beginning with 4 excludes a patient. Chest compressions are identified by prehospital procedure code 6 or emergency circulatory procedure code 1/2. Repeated entries of the same target code are not separate patients.
